# Mol­ecular and crystal structure of gossypol tetra­methyl ether with an unknown solvate

**DOI:** 10.1107/S2056989015000171

**Published:** 2015-01-21

**Authors:** Muhabbat Honkeldieva, Samat Talipov, Rustam Mardanov, Bakhtiyar Ibragimov

**Affiliations:** a Institute of Bioorganic Chemistry, Mirzo-Ulughbek Str. 83, Tashkent, 100125, Uzbekistan

**Keywords:** crystal structure, gossypol, gossypol tetra­methyl ether, porous structure, C—H⋯O hydrogen bonds, C—H⋯π inter­actions

## Abstract

The title compound, C_34_H_38_O_8_ (systematic name: 5,5′-diisopropyl-2,2′,3,3′-tetra­meth­oxy-7,7′-dimethyl-2*H*,2′*H*-8,8′-bi­[naphtho­[1,8-*bc*]furan]-4,4′-diol), has been obtained from a gossypol solution in a mixture of dimethyl sulfate and methanol. The mol­ecule is situated on a twofold rotation axis, so the asymmetric unit contains one half-mol­ecule. In the mol­ecule, the hy­droxy groups are involved in intra­molecular O—H⋯O hydrogen bonds, and the two naphthyl fragments are inclined each to other by 83.8 (1)°. In the crystal, weak C—H⋯O and C—H⋯π inter­actions consolidate the packing, which exhibits channels with an approximate diameter of 6 Å extending along the *c*-axis direction. These channels are filled with highly disordered solvent mol­ecules, so their estimated scattering contribution was subtracted from the observed diffraction data using the SQUEEZE option in *PLATON* [Spek, A. L. (2015). *Acta Cryst*. C**71**, 9–18].

## Abstract 2

The title compound consists of two planar halves. There is one half-mol­ecule in the asymmetric unit, the whole mol­ecule being generated by twofold rotation symmetry. The crystal structure has wide channels of 5–6 Å in diameter extending along the *c*-axis direction. The mol­ecules are associated into a three-dimensional network supported by some weak C—H⋯O hydrogen bonds and C—H⋯π inter­actions.

## Chemical context   

Gossypol [systematic name: 2,2′-bis­(8-formyl-1,6,7-tri­hydroxyl-5-isopropyl-3-methyl­naphthalene)] is an unique terpenoid found in *Gossypium* (cotton) and related species. Within plants, gossypol appears to act as a natural insecticide and fungicide (Adams *et al.*, 1960 ▸). Because of its anti­nutritive effect, gossypol limits the feeding of cottonseed and cottonseed meal to ruminant animals. However, the compound also has a wide range of biological actions, including anti-HIV, anti­cancer, and anti­fertility effects (Liang *et al.*, 1995 ▸; Dorsett *et al.*, 1975 ▸; Coutinho, 2002 ▸; Royer *et al.*, 1995 ▸). Gossypol is a surprisingly versatile host compound that forms inclusion complexes with a great variety of organic substances such as ketones, ethers, esters, organic and mineral acids, water, various benzyl compounds and chlorinated and brominated compounds. More than one hundred of these complexes with different guest mol­ecules have been obtained and structurally characterized (Talipov *et al.*, 2002 ▸; 2003 ▸; 2007 ▸; Ibragimov *et al.*, 2004 ▸). A specific feature of gossypol is the existence of gossypol host–guest complexes in the form of polymorphic crystals. As a result of its comprehensive biological properties, there is current inter­est in the synthesis of new gossypol derivatives. Many derivatives have been reported, including ethers, acetates and Schiff bases with aldehydes (Talipov *et al.*, 2004 ▸; 2009 ▸; Tilyabaev *et al.*, 2009 ▸; Kenar, 2006 ▸). As first reported by Morris & Adams (1937 ▸), treatment with an alkali of a gossypol solution in a mixture of dimethyl sulfate and methanol, yields a white gossypol tetra­methyl ether, the title compound.
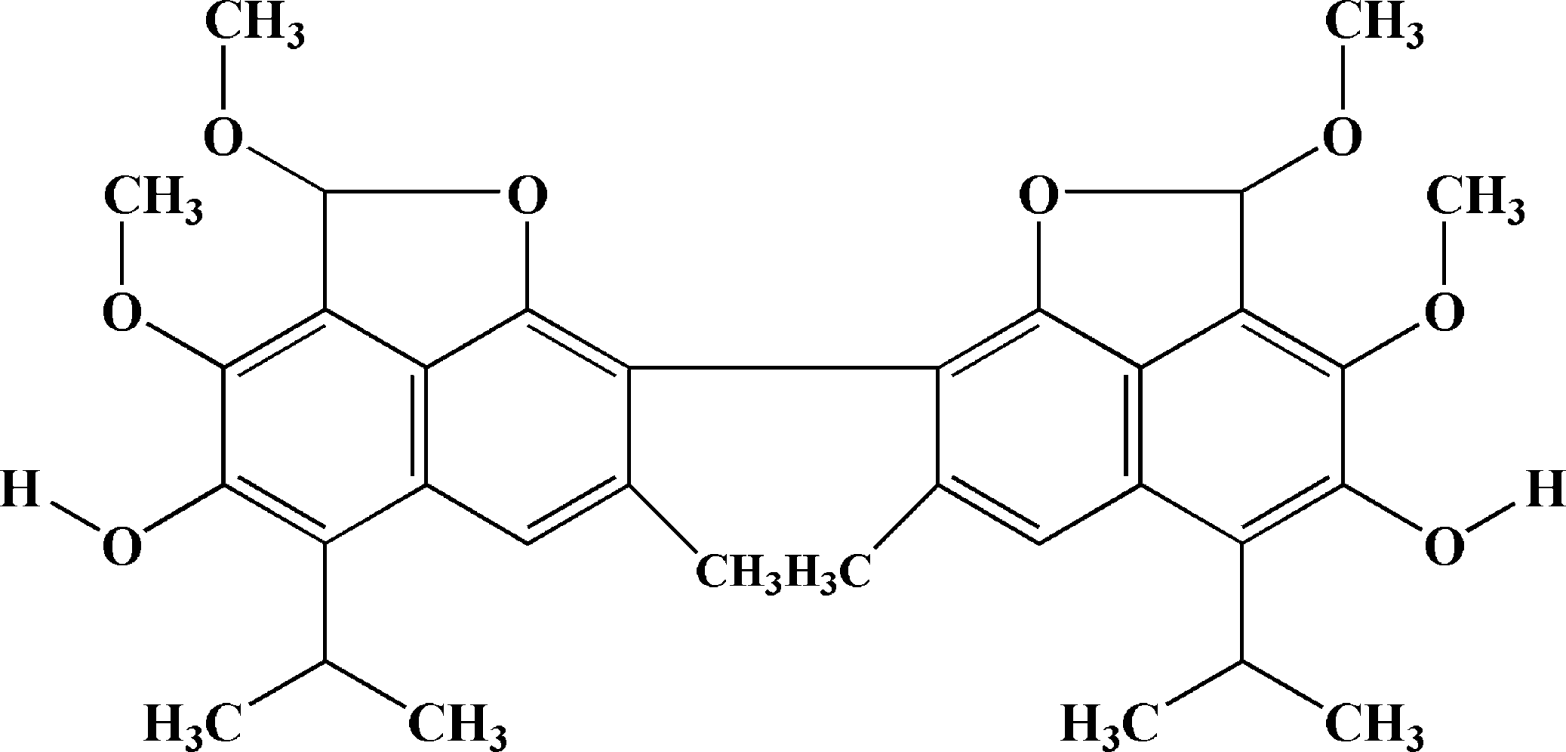


## Structural commentary   

Gossypol can exist in one of the following tautomeric forms: aldehyde, quinoid and lactol (Adams *et al.*, 1960 ▸). In most solvents it is found in the aldehyde form. However, there are some reports that gossypol also exists in a pure lactol form (Reyes *et al.*, 1986 ▸) or as a dynamic equilibrium mixture of the aldehyde and lactol forms in some highly polar solvents (Kamaev *et al.*, 1979 ▸). In the structure described here, the title compound exists in the lactol form.

**Figure 1:**
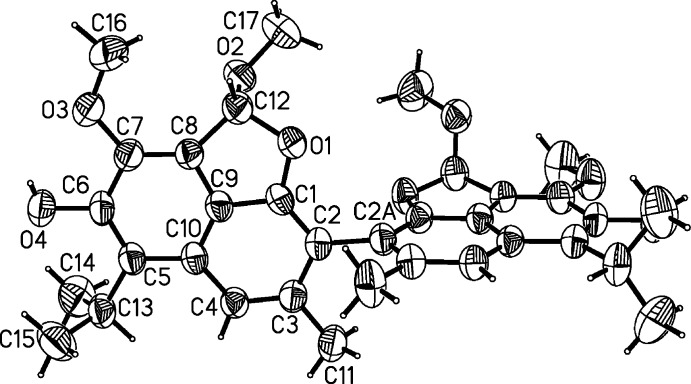
The mol­ecular structure of the title compound showing the atomic numbering and 50% probability displacement ellipsoids. Unlabeled atoms are related to labeled ones by the symmetry operation (A) −*x*, *y*, 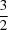 − *z*.

The crystallographically imposed symmetry of the title mol­ecule is *C*2; the twofold axis is perpendicular to the C2—C2*A* bond [symmetry code (*A*): −*x*, *y*, 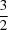 − *z*]. The symmetry of the mol­ecule corresponds to symmetry of the crystal, the title compound mol­ecule being situated on a twofold axis. An *ORTEP* diagram of the mol­ecule showing the atom-numbering scheme is given in Fig. 1 ▸. The mol­ecule consists of two fused ring systems, each containing a naphthalene ring system with a fused furan ring. The two napthyl bicycles of the mol­ecule are nearly perpendicular and the dihedral angle between their least-squares planes is 83.8 (1)°. The furan ring is not completely planar, with atom C12 deviating from the C1/O1/C8/C9 plane by 0.225 (4) Å. The meth­oxy group at the C-7 position is almost coplanar with the plane of the naphthalene ring system; atomic deviations from this plane are 0.004 (3) for O3 and 0.163 (5) Å for C16. The meth­oxy group on the furan ring (C12-O2-C17H_3_) and atom O1 are located on the same side of the host ring (C1–C4/C9/C10). The isopropyl groups are positioned with the ternary hydrogen atoms pointed outwards and away from the center of the mol­ecule, the isopropyl groups bis­ect the extended naphthalene ring system plane.

There is an intra­molecular O4—H4⋯O3 hydrogen bond (Table 1 ▸) which is similar to those observed previously in structures of gossypol and its Schiff bases. The values of the bond lengths and angles in the title mol­ecule are within expected values. However, there are notable differences in the lengths of some of these bonds compared with typical values for gossypol structures. Compared with the relatively short C5—C6 aromatic ring bonds of gossypol mol­ecules (1.36 Å), the corresponding bond in the title mol­ecule is longer at 1.380 (3) Å. In addition, the C7—C8 and C8—C9 bonds in the title compound are shorter than those in gossypol by 0.03 and 0.06 Å, respectively. The shortest bond within these rings is the C1–C2 bond with a length of 1.359 (3) Å. In the furan ring, there are some differences in the lengths of some bonds compared with the values found in dianhydro­gossypol. In the title mol­ecule, the C1—O1 bond [1.374 (3) Å] is shorter than the O1—C12 bond [1.463 (3) Å].

## Supra­molecular features   

**Figure 2:**
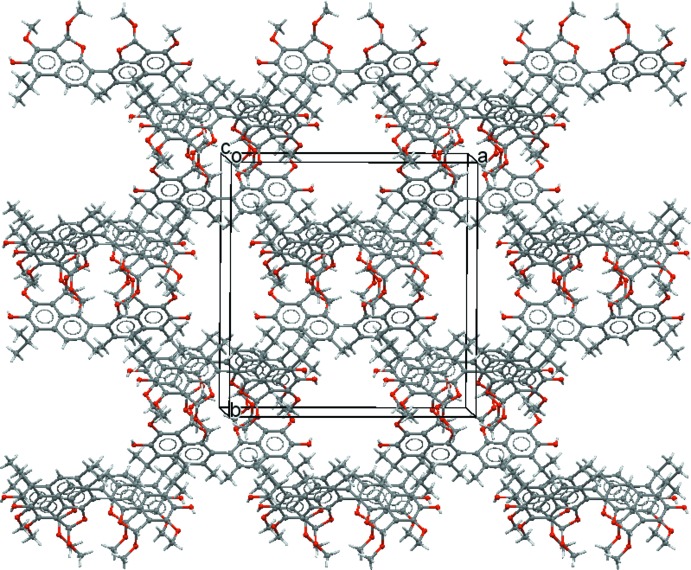
A portion of the crystal packing viewed approximately along the *c* axis.

The packing of the title mol­ecules is shown in Fig. 2 ▸. Weak inter­molecular C—H⋯O and C—H⋯π inter­actions (Table 1 ▸) consolidate the crystal packing, which exhibits channels with a diameter of approximately 6 Å extending along the *c*-axis direction. These channels are similar to the channels previously reported in a dianhydro­gossypol crystal structure (Talipov *et al.*, 2009 ▸). In the present structure, for each unit cell, the channels provide a void volume of 672 Å^3^ corres­ponding to 19% of the unit-cell volume. Highly disordered solvent mol­ecules, most probably water mol­ecules, occupy these voids in the crystal; their contribution to the scattering was removed with the SQUEEZE routine of the *PLATON* program (Spek, 2009 ▸, 2015 ▸).

## Database survey   

A search in the Cambridge Structural Database (Version 5.33, last update November 2013; Groom & Allen, 2014 ▸) indicated the presence of 191 entries for gossypol (137 entries) or gossypol derivatives. However, only four entries were found for fused-ring systems containing a naphthalene ring system with a fused furan ring. The dihedral angle between two fused ring systems in these structures is equal to 84.8 in TEYJEM (Ibragimov *et al.*, 1995 ▸), 111.8 in TEYJEN (Ibragimov *et al.*, 1995 ▸), 117.0 in YURMEE (Talipov *et al.*, 1999 ▸) and 119.1° in FOVKEG (Talipov *et al.*, 1999 ▸).

## Synthesis and crystallization   

Gossypol was obtained from the Experimental Plant of the Institute of Bioorganic Chemistry, Academy of Sciences of Uzbekistan where it was produced from by-products of the cottonseed oil industry. The title compound was synthesized following the known procedure (Morris & Adams, 1937 ▸). In order to prepare single crystals suitable for X-ray experiments, powdered material was dissolved in acetone (20 mg/1 ml) and stored for few days at room temperature under slow evaporation of the solution.

## Refinement   

Crystal data, data collection and structure refinement details are summarized in Table 2 ▸. The H atom of the hydroxyl substituent was located in an electron density map and its coordinates were freely refined with *U_iso_* = 1.5*U*_eq_(O). C-bound H atoms were positioned geometrically and refined using a riding model, with *d*(C—H) = 0.93 Å and *U_iso_* = 1.2*U_eq_* (C) for aromatic, *d*(C—H) = 0.98 Å and *U_iso_* = 1.2*U_eq_* (C) for methine, *d*(C—H) = 0.96 Å and *U_iso_* = 1.5*U_eq_* (C) for methyl H atoms.

## Supplementary Material

Crystal structure: contains datablock(s) I. DOI: 10.1107/S2056989015000171/cv5482sup1.cif

Structure factors: contains datablock(s) I. DOI: 10.1107/S2056989015000171/cv5482Isup2.hkl

CCDC reference: 1007641

Additional supporting information:  crystallographic information; 3D view; checkCIF report

## Figures and Tables

**Table 1:** Hydrogen-bond geometry (, ) *Cg* is the centroid of the C1C4/C9/C10 ring.

*D*H*A*	*D*H	H*A*	*D* *A*	*D*H*A*
O4H4O3	0.67(3)	2.17(4)	2.586(3)	122(4)
C17H17*A*O1^i^	0.96	2.71	3.286(3)	119
C17H17*C* *Cg* ^ii^	0.96	2.77	3.551(4)	139

Symmetry codes: (i) 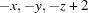; (ii) 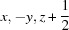.

**Table 2:** Experimental details

Crystal data	
Chemical formula	C_34_H_38_O_8_
*M*_r_	574.64
Crystal system, space group	Orthorhombic, *P* *b* *c* *n*
Temperature (K)	293
*a*, *b*, *c* ()	19.7086(5), 20.3099(7), 8.8443(4)
*V* (^3^)	3540.2(2)
*Z*	4
Radiation type	Cu *K*
(mm^1^)	0.62
Crystal size (mm)	0.35 0.28 0.26

Data collection	
Diffractometer	Oxford Diffraction Xcalibur Ruby
Absorption correction	Multi-scan (SCALE3 ABSPACK in *CrysAlis PRO*; Oxford Diffraction, 2009 ▸)
*T*_min_, *T*_max_	0.914, 1.000
No. of measured, independent and observed [*I* > 2(*I*)] reflections	12345, 3340, 1826
*R*_int_	0.049
(sin /)_max_ (^1^)	0.613

Refinement	
*R*[*F* ^2^ > 2(*F* ^2^)], *wR*(*F* ^2^), *S*	0.056, 0.162, 0.93
No. of reflections	3340
No. of parameters	200
H-atom treatment	H atoms treated by a mixture of independent and constrained refinement
_max_, _min_ (e ^3^)	0.24, 0.17

Computer programs: *CrysAlis PRO* (Oxford Diffraction, 2009 ▸), *SHELXS97*, *SHELXL97* and *XP* in *SHELXTL* (Sheldrick, 2008 ▸).

## supplementary crystallographic information

### Crystal data

**Table:**

C_34_H_38_O_8_	*D*_x_ = 1.078 Mg m^−^^3^
*M**_r_* = 574.64	Cu *K*α radiation, λ = 1.54184 Å
Orthorhombic, *P**b**c**n*	Cell parameters from 2468 reflections
*a* = 19.7086 (5) Å	θ = 4.4–70.6°
*b* = 20.3099 (7) Å	µ = 0.62 mm^−^^1^
*c* = 8.8443 (4) Å	*T* = 293 K
*V* = 3540.2 (2) Å^3^	Prism, white
*Z* = 4	0.35 × 0.28 × 0.26 mm
*F*(000) = 1224	

### Data collection

**Table:**

Oxford Diffraction Xcalibur Ruby diffractometer	3340 independent reflections
Radiation source: fine-focus sealed tube	1826 reflections with *I* > 2σ(*I*)
Graphite monochromator	*R*_int_ = 0.049
Detector resolution: 10.2576 pixels mm^-1^	θ_max_ = 71.0°, θ_min_ = 4.4°
ω scans	*h* = −23→24
Absorption correction: multi-scan (SCALE3 ABSPACK in *CrysAlis PRO*; Oxford Diffraction, 2009)	*k* = −22→24
*T*_min_ = 0.914, *T*_max_ = 1.000	*l* = −10→10
12345 measured reflections	

### Refinement

**Table:**

Refinement on *F*^2^	Secondary atom site location: difference Fourier map
Least-squares matrix: full	Hydrogen site location: inferred from neighbouring sites
*R*[*F*^2^ > 2σ(*F*^2^)] = 0.056	H atoms treated by a mixture of independent and constrained refinement
*wR*(*F*^2^) = 0.162	*w* = 1/[σ^2^(*F*_o_^2^) + (0.0932*P*)^2^] where *P* = (*F*_o_^2^ + 2*F*_c_^2^)/3
*S* = 0.93	(Δ/σ)_max_ < 0.001
3340 reflections	Δρ_max_ = 0.24 e Å^−^^3^
200 parameters	Δρ_min_ = −0.17 e Å^−^^3^
0 restraints	Extinction correction: *SHELXL*, Fc^*^=kFc[1+0.001xFc^2^λ^3^/sin(2θ)]^-1/4^
Primary atom site location: structure-invariant direct methods	Extinction coefficient: 0.00096 (17)

### Special details

**Table:**

Geometry. All e.s.d.'s (except the e.s.d. in the dihedral angle between two l.s. planes) are estimated using the full covariance matrix. The cell e.s.d.'s are taken into account individually in the estimation of e.s.d.'s in distances, angles and torsion angles; correlations between e.s.d.'s in cell parameters are only used when they are defined by crystal symmetry. An approximate (isotropic) treatment of cell e.s.d.'s is used for estimating e.s.d.'s involving l.s. planes.
Refinement. Refinement of *F*^2^ against ALL reflections. The weighted *R*-factor *wR* and goodness of fit *S* are based on *F*^2^, conventional *R*-factors *R* are based on *F*, with *F* set to zero for negative *F*^2^. The threshold expression of *F*^2^ > σ(*F*^2^) is used only for calculating *R*-factors(gt) *etc*. and is not relevant to the choice of reflections for refinement. *R*-factors based on *F*^2^ are statistically about twice as large as those based on *F*, and *R*- factors based on ALL data will be even larger.

### Fractional atomic coordinates and isotropic or equivalent isotropic displacement parameters (Å^2^)

**Table:**

	*x*	*y*	*z*	*U*_iso_*/*U*_eq_	
O1	0.06600 (7)	0.08142 (8)	0.91284 (19)	0.0592 (5)	
O2	0.12998 (9)	−0.01420 (8)	0.8665 (2)	0.0664 (5)	
O3	0.29694 (8)	0.05026 (10)	0.9636 (2)	0.0735 (6)	
O4	0.35682 (9)	0.13669 (12)	0.7953 (3)	0.0731 (6)	
C1	0.08110 (10)	0.12972 (12)	0.8095 (3)	0.0487 (6)	
C2	0.03731 (10)	0.17024 (12)	0.7358 (3)	0.0486 (6)	
C3	0.06794 (11)	0.21591 (12)	0.6321 (3)	0.0521 (6)	
C4	0.13705 (10)	0.22063 (12)	0.6150 (2)	0.0503 (6)	
H4A	0.1545	0.2512	0.5472	0.060*	
C5	0.25535 (11)	0.18076 (12)	0.6976 (2)	0.0498 (6)	
C6	0.28733 (10)	0.13639 (12)	0.7919 (3)	0.0518 (6)	
C7	0.25350 (11)	0.08978 (12)	0.8849 (3)	0.0532 (6)	
C8	0.18412 (11)	0.08864 (11)	0.8835 (2)	0.0482 (6)	
C9	0.15172 (10)	0.13466 (11)	0.7923 (2)	0.0465 (5)	
C10	0.18240 (10)	0.18039 (12)	0.6973 (2)	0.0463 (6)	
C11	0.02224 (12)	0.25791 (16)	0.5367 (3)	0.0750 (9)	
H11B	0.0492	0.2873	0.4766	0.113*	
H11C	−0.0073	0.2830	0.6009	0.113*	
H11A	−0.0044	0.2303	0.4716	0.113*	
C12	0.12826 (11)	0.04508 (13)	0.9467 (3)	0.0575 (7)	
H12	0.1337	0.0379	1.0556	0.069*	
C13	0.29419 (11)	0.22682 (14)	0.5945 (3)	0.0618 (7)	
H13	0.2602	0.2522	0.5385	0.074*	
C14	0.33502 (14)	0.18881 (18)	0.4773 (3)	0.0909 (10)	
H14A	0.3052	0.1608	0.4204	0.136*	
H14C	0.3686	0.1624	0.5273	0.136*	
H14B	0.3570	0.2192	0.4102	0.136*	
C15	0.33765 (15)	0.27649 (16)	0.6781 (4)	0.0922 (11)	
H15B	0.3096	0.3020	0.7448	0.138*	
H15A	0.3592	0.3052	0.6065	0.138*	
H15C	0.3716	0.2538	0.7357	0.138*	
C16	0.26946 (16)	0.00777 (18)	1.0766 (4)	0.1007 (12)	
H16C	0.3057	−0.0154	1.1260	0.151*	
H16A	0.2393	−0.0233	1.0301	0.151*	
H16B	0.2450	0.0334	1.1496	0.151*	
C17	0.08064 (15)	−0.06108 (16)	0.9165 (4)	0.0945 (11)	
H17B	0.0860	−0.1014	0.8610	0.142*	
H17A	0.0359	−0.0438	0.8997	0.142*	
H17C	0.0869	−0.0695	1.0224	0.142*	
H4	0.3667 (18)	0.1176 (19)	0.854 (4)	0.103 (15)*	

### Atomic displacement parameters (Å^2^)

**Table:**

	*U*^11^	*U*^22^	*U*^33^	*U*^12^	*U*^13^	*U*^23^
O1	0.0420 (8)	0.0675 (11)	0.0681 (11)	0.0003 (8)	0.0123 (8)	0.0175 (9)
O2	0.0564 (10)	0.0584 (11)	0.0845 (13)	−0.0061 (8)	0.0122 (9)	0.0081 (10)
O3	0.0514 (10)	0.0864 (14)	0.0826 (13)	0.0050 (9)	−0.0047 (9)	0.0296 (11)
O4	0.0407 (10)	0.0939 (16)	0.0847 (15)	−0.0017 (9)	−0.0052 (9)	0.0252 (13)
C1	0.0415 (12)	0.0552 (14)	0.0493 (13)	−0.0065 (10)	0.0090 (10)	0.0020 (12)
C2	0.0375 (11)	0.0582 (14)	0.0501 (13)	0.0007 (11)	0.0027 (10)	−0.0023 (12)
C3	0.0425 (12)	0.0617 (15)	0.0519 (13)	0.0020 (11)	0.0005 (10)	0.0027 (12)
C4	0.0432 (12)	0.0590 (15)	0.0487 (13)	−0.0012 (11)	0.0035 (10)	0.0063 (11)
C5	0.0394 (11)	0.0610 (15)	0.0489 (13)	−0.0040 (10)	0.0019 (10)	0.0022 (12)
C6	0.0328 (11)	0.0673 (16)	0.0553 (13)	−0.0021 (11)	−0.0013 (10)	0.0006 (12)
C7	0.0454 (12)	0.0613 (16)	0.0530 (14)	0.0030 (11)	−0.0028 (11)	0.0063 (12)
C8	0.0412 (12)	0.0544 (14)	0.0491 (13)	0.0001 (10)	0.0029 (10)	0.0052 (11)
C9	0.0402 (11)	0.0545 (14)	0.0448 (12)	−0.0016 (10)	0.0042 (10)	0.0000 (11)
C10	0.0393 (11)	0.0569 (14)	0.0428 (12)	−0.0033 (10)	0.0037 (9)	−0.0035 (11)
C11	0.0463 (13)	0.094 (2)	0.085 (2)	0.0059 (14)	−0.0003 (13)	0.0254 (17)
C12	0.0486 (13)	0.0697 (17)	0.0543 (15)	0.0002 (12)	0.0060 (11)	0.0100 (13)
C13	0.0400 (12)	0.0816 (18)	0.0638 (16)	−0.0047 (12)	0.0038 (11)	0.0176 (14)
C14	0.0742 (19)	0.124 (3)	0.075 (2)	0.0012 (18)	0.0247 (16)	0.020 (2)
C15	0.086 (2)	0.097 (2)	0.094 (2)	−0.0331 (19)	−0.0049 (17)	0.024 (2)
C16	0.077 (2)	0.119 (3)	0.106 (2)	0.002 (2)	−0.0097 (18)	0.062 (2)
C17	0.084 (2)	0.081 (2)	0.119 (3)	−0.0242 (18)	0.0142 (19)	0.022 (2)

### Geometric parameters (Å, º)

**Table:**

O1—C1	1.374 (3)	C8—C12	1.519 (3)
O1—C12	1.463 (3)	C9—C10	1.391 (3)
O2—C12	1.398 (3)	C11—H11B	0.9600
O2—C17	1.431 (3)	C11—H11C	0.9600
O3—C7	1.364 (3)	C11—H11A	0.9600
O3—C16	1.427 (3)	C12—H12	0.9800
O4—C6	1.370 (3)	C13—C15	1.516 (4)
O4—H4	0.67 (3)	C13—C14	1.523 (4)
C1—C2	1.359 (3)	C13—H13	0.9800
C1—C9	1.404 (3)	C14—H14A	0.9600
C2—C3	1.437 (3)	C14—H14C	0.9600
C2—C2^i^	1.492 (4)	C14—H14B	0.9600
C3—C4	1.374 (3)	C15—H15B	0.9600
C3—C11	1.500 (3)	C15—H15A	0.9600
C4—C10	1.413 (3)	C15—H15C	0.9600
C4—H4A	0.9300	C16—H16C	0.9600
C5—C6	1.380 (3)	C16—H16A	0.9600
C5—C10	1.438 (3)	C16—H16B	0.9600
C5—C13	1.514 (3)	C17—H17B	0.9600
C6—C7	1.420 (3)	C17—H17A	0.9600
C7—C8	1.368 (3)	C17—H17C	0.9600
C8—C9	1.390 (3)		
			
C1—O1—C12	108.35 (16)	H11B—C11—H11A	109.5
C12—O2—C17	113.6 (2)	H11C—C11—H11A	109.5
C7—O3—C16	118.3 (2)	O2—C12—O1	110.5 (2)
C6—O4—H4	108 (3)	O2—C12—C8	107.31 (18)
C2—C1—O1	127.89 (19)	O1—C12—C8	103.82 (18)
C2—C1—C9	122.3 (2)	O2—C12—H12	111.6
O1—C1—C9	109.76 (19)	O1—C12—H12	111.6
C1—C2—C3	115.48 (19)	C8—C12—H12	111.6
C1—C2—C2^i^	123.0 (2)	C5—C13—C15	113.8 (2)
C3—C2—C2^i^	121.44 (19)	C5—C13—C14	111.3 (2)
C4—C3—C2	122.1 (2)	C15—C13—C14	111.8 (2)
C4—C3—C11	119.6 (2)	C5—C13—H13	106.5
C2—C3—C11	118.3 (2)	C15—C13—H13	106.5
C3—C4—C10	122.0 (2)	C14—C13—H13	106.5
C3—C4—H4A	119.0	C13—C14—H14A	109.5
C10—C4—H4A	119.0	C13—C14—H14C	109.5
C6—C5—C10	117.0 (2)	H14A—C14—H14C	109.5
C6—C5—C13	122.44 (19)	C13—C14—H14B	109.5
C10—C5—C13	120.5 (2)	H14A—C14—H14B	109.5
O4—C6—C5	117.8 (2)	H14C—C14—H14B	109.5
O4—C6—C7	117.3 (2)	C13—C15—H15B	109.5
C5—C6—C7	124.81 (19)	C13—C15—H15A	109.5
O3—C7—C8	128.5 (2)	H15B—C15—H15A	109.5
O3—C7—C6	113.13 (19)	C13—C15—H15C	109.5
C8—C7—C6	118.4 (2)	H15B—C15—H15C	109.5
C7—C8—C9	116.9 (2)	H15A—C15—H15C	109.5
C7—C8—C12	137.0 (2)	O3—C16—H16C	109.5
C9—C8—C12	105.79 (18)	O3—C16—H16A	109.5
C8—C9—C10	126.9 (2)	H16C—C16—H16A	109.5
C8—C9—C1	110.1 (2)	O3—C16—H16B	109.5
C10—C9—C1	123.0 (2)	H16C—C16—H16B	109.5
C9—C10—C4	114.98 (19)	H16A—C16—H16B	109.5
C9—C10—C5	115.9 (2)	O2—C17—H17B	109.5
C4—C10—C5	129.1 (2)	O2—C17—H17A	109.5
C3—C11—H11B	109.5	H17B—C17—H17A	109.5
C3—C11—H11C	109.5	O2—C17—H17C	109.5
H11B—C11—H11C	109.5	H17B—C17—H17C	109.5
C3—C11—H11A	109.5	H17A—C17—H17C	109.5
			
C12—O1—C1—C2	172.7 (2)	C7—C8—C9—C1	−176.7 (2)
C12—O1—C1—C9	−9.9 (3)	C12—C8—C9—C1	8.3 (3)
O1—C1—C2—C3	−179.4 (2)	C2—C1—C9—C8	178.3 (2)
C9—C1—C2—C3	3.6 (3)	O1—C1—C9—C8	0.8 (3)
O1—C1—C2—C2^i^	3.4 (4)	C2—C1—C9—C10	−1.0 (4)
C9—C1—C2—C2^i^	−173.7 (2)	O1—C1—C9—C10	−178.6 (2)
C1—C2—C3—C4	−3.4 (3)	C8—C9—C10—C4	178.9 (2)
C2^i^—C2—C3—C4	173.9 (2)	C1—C9—C10—C4	−1.9 (3)
C1—C2—C3—C11	174.6 (2)	C8—C9—C10—C5	−1.7 (3)
C2^i^—C2—C3—C11	−8.1 (4)	C1—C9—C10—C5	177.5 (2)
C2—C3—C4—C10	0.6 (4)	C3—C4—C10—C9	2.0 (3)
C11—C3—C4—C10	−177.4 (2)	C3—C4—C10—C5	−177.2 (2)
C10—C5—C6—O4	−179.0 (2)	C6—C5—C10—C9	−0.4 (3)
C13—C5—C6—O4	2.7 (4)	C13—C5—C10—C9	177.8 (2)
C10—C5—C6—C7	1.6 (4)	C6—C5—C10—C4	178.8 (2)
C13—C5—C6—C7	−176.6 (2)	C13—C5—C10—C4	−2.9 (4)
C16—O3—C7—C8	−10.0 (4)	C17—O2—C12—O1	−68.7 (3)
C16—O3—C7—C6	171.4 (2)	C17—O2—C12—C8	178.7 (2)
O4—C6—C7—O3	−1.3 (3)	C1—O1—C12—O2	−100.5 (2)
C5—C6—C7—O3	178.0 (2)	C1—O1—C12—C8	14.3 (2)
O4—C6—C7—C8	179.9 (2)	C7—C8—C12—O2	−70.0 (4)
C5—C6—C7—C8	−0.8 (4)	C9—C8—C12—O2	103.5 (2)
O3—C7—C8—C9	−179.8 (2)	C7—C8—C12—O1	172.9 (3)
C6—C7—C8—C9	−1.3 (4)	C9—C8—C12—O1	−13.6 (2)
O3—C7—C8—C12	−6.8 (5)	C6—C5—C13—C15	−64.3 (3)
C6—C7—C8—C12	171.7 (3)	C10—C5—C13—C15	117.5 (3)
C7—C8—C9—C10	2.6 (4)	C6—C5—C13—C14	63.1 (3)
C12—C8—C9—C10	−172.4 (2)	C10—C5—C13—C14	−115.1 (3)

Symmetry code: (i) −*x*, *y*, −*z*+3/2.

### Hydrogen-bond geometry (Å, º)

Cg is the centroid of the C1–C4/C9/C10 ring.

**Table:**

*D*—H···*A*	*D*—H	H···*A*	*D*···*A*	*D*—H···*A*
O4—H4···O3	0.67 (3)	2.17 (4)	2.586 (3)	122 (4)
C17—H17*A*···O1^ii^	0.96	2.71	3.286 (3)	119
C17—H17*C*···*Cg*^iii^	0.96	2.77	3.551 (4)	139

Symmetry codes: (ii) −*x*, −*y*, −*z*+2; (iii) *x*, −*y*, *z*+1/2.
